# Trends in phase III randomized controlled clinical trials on the treatment of advanced non‐small‐cell lung cancer

**DOI:** 10.1002/cam4.782

**Published:** 2016-07-23

**Authors:** Cristina Fernández‐López, José Expósito‐Hernández, Juan Pedro Arrebola‐Moreno, Miguel Ángel Calleja‐Hernández, Manuela Expósito‐Ruíz, Rosa Guerrero‐Tejada, Isabel Linares, José Cabeza‐Barrera

**Affiliations:** ^1^ Department of PharmacyBiosanitary Institute of Granada (ibs.GRANADA)University Hospitals of Granada/University of GranadaGranadaSpain; ^2^ Department of OncologyVirgen de las Nieves Universitary HospitalGranadaSpain; ^3^ Unit Research SupportBiosanitary Institute of Granada (ibs.GRANADA)University Hospitals of Granada/University of GranadaGranadaSpain

**Keywords:** Advanced stage, non‐small‐cell lung cancer, randomized controlled trial, review, treatment

## Abstract

The objective of this review was to analyze trends in outcomes and in the quality of phase III randomized controlled trials on advanced NSCLC published between 2000 and 2012, selecting 76 trials from a total of 122 retrieved in a structured search. Over the study period, the number of randomized patients per trial increased by 14 per year (*P *=* *0.178). The sample size significantly increased between 2000 and 2012 in trials of targeted agents (460.1 vs. 740.8 patients, *P *=* *0.009), trials of >1 drug (360.4 vs. 584.8, *P *=* *0.014), and those including patients with good performance status (675.3 vs. 425.6; *P *=* *0.003). Quality of life was assessed in 46 trials (60.5%), and significant improvements were reported in 10 of these (21.7%). Platinum‐based regimens were the most frequently investigated (86.8% of trials). Molecular‐targeted agents were studied in 25.0% of chemotherapy arms, and the percentage of trials including these agents increased each year. The median (interquartile range) overall survival (MOS) was 9.90 (3.5) months with an increase of 0.384 months per year of publication (*P *<* *0.001). A statistically significant improvement in MOS was obtained in only 13 (18.8%) trials. The median progression‐free survival was 4.9 (1.9) months, with a nonsignificant increase of 0.026 months per year (*P *>* *0.05). There has been a continuous but modest improvement in the survival of patients with advanced NSCLC over the past 12 years. Nevertheless, the quality of clinical trials and the benefit in outcomes should be carefully considered before the incorporation of novel approaches into clinical practice.

## Introduction

Lung cancer has been the most common cancer worldwide for several decades 1, 2, with 1.8 million estimated new cases in 2012, 12.9% of the total. It has also been the most frequent cause of death from cancer, being responsible for nearly one in five instances (1.59 million deaths, 19.4% of total) 3. Non‐small‐cell lung cancer (NSCLC) represents 85–90% of lung cancer cases 4, and 40% of patients with newly diagnosed NSCLC have stage IV disease, with a median survival ranging from 3 to 15 months 5 and an estimated 5‐year relative survival rate of <18% 6, 7. Current treatment goals are to prolong survival and control disease‐related symptoms.

Numerous clinical trials have been conducted over recent decades to identify the optimal therapeutic option for patients with NSCLC. Although cytotoxic chemotherapy has been the main treatment, novel agents against specific molecules are under investigation for selected histologic subgroups (e.g., erlotinib, which targets the epidermal growth factor receptor (EGFR) and crizotinib, which targets the EML4/ALK molecular pathway) 8, 9, 10, 11. However, only modest clinical benefits have been achieved despite these novel approaches and the 5‐year relative survival rate in lung cancer has shown the least improvement among all cancers over the past three decades 12. In spite of major research efforts, improving the survival and quality of life (QoL) of lung cancer patients remains one of the greatest challenges in oncology 13.

Phase III randomized clinical trials (RCTs) are the main tool for testing new anti‐cancer agents 14. The increasing number of RCTs on the effectiveness of chemotherapy against NSCLC over recent decades indicates the growing interest of the oncology community and pharmaceutical industry in this entity 10, 15. However, the results have been inconsistent.

Although the design of RCTs in oncology has improved over time, they still suffer from shortcomings that may have a major impact on the conclusions drawn from their results 14. The aim of the present review of published RCTs on NSCLC was to analyze changes in design quality and in the efficacy of the treatments tested over the past 12 years.

## Methods

### Literature search strategy and study selection criteria

A structured search was conducted for randomized phase III RCTs reported between 2000 and 2012 on the utilization of cytotoxic agents or molecular‐targeted agents in patients with advanced NSCLC. MEDLINE (access via PubMed and Ovid SP), EMBASE (Ovid SP), CENTRAL (Cochrane Central Register of Controlled Trials, Cochrane Library), Evidence‐Cancer (via NHS), and TRIP (via BV‐SSPA) were searched using the following search terms: lung cancer, non‐small, advanced, non‐surgical, treatment, controlled clinical trials, and phase III.

The inclusion criterion was the comparison of at least two arms of systemic chemotherapy or molecular‐targeted agents in patients with advanced NSCLC. We also considered RCTs publishing interim findings and those comparing different dosage regimens of the same agent or combination of agents. In trials with two or more experimental arms, the arm selected for evaluation in this review was that which obtained the highest overall survival (OS) or progression‐free survival, depending on the primary outcome of the trial. Only RCTs published in journals were selected.

Exclusion criteria were: limitation of the RCT to nonadvanced disease or to any stage of small‐cell lung cancer; studies of surgical or radiotherapy intervention or of cancer screening and prevention; meta‐analyses or reviews reporting data from multiple RCTs; preliminary studies for which subsequent phase III studies were available; retrospective series; studies presenting results for a subgroup of the original study population; RCTs in which immunotherapy regimens alone were investigated or in which the compared arms used the same chemotherapy regimen and only differed in the non‐chemotherapeutic agent(s) administered; congress abstracts; and studies in a language other than English.

### Methodology

Selected trials were scrutinized to identify potential duplication or overlap. Two reviewers (radiation oncologist with expertise in lung cancer [J.E.‐H.] and clinical pharmacist [C.F.‐L.]) independently conducted all study selection and data extraction procedures to minimize bias. They independently checked the references obtained by structured searching and then obtained all data from selected studies, independently assessing them for internal consistency and comparing their results. Disagreements were resolved by discussion between the investigators to reach a consensus.

### Data extraction and management

Data were extracted from each selected publication using a specifically designed form to record the following information: publication year, first author, study sponsorship (based on explicit statements in the article and author affiliations), journal and impact factor (IF), sample size, stage of the disease, performance status of patients (mainly according to ECOG grade or Karnofsky Performance Status [KPS] scale), treatment line (first or second), chemotherapy regimen (agents, dose, and administration scheme), number of agents combined, median cycles administered per arm, median follow‐up, intention to treat (ITT) analysis (based on raw data in the article), assessment of QoL, median overall survival (MOS, months), objective response rate (%), median progression‐free survival (MPFS, months) per arm, and any statistically significant differences observed between therapeutic arms. We also recorded the authors’ conclusions on the experimental arm, summarized as a positive result, negative result, or similar result for experimental versus control arms. Data from studies published in multiple journals were extracted and reported as a single study.

### Statistical analyses

In a first descriptive analysis, relative and absolute frequencies were calculated for qualitative variables and tendency and dispersion measures for numerical variables. Bivariate analyses were then conducted on the association between variables and the year of publication, using the Mann–Whitney U test and applying Pearson/Spearman correlations when appropriate. *P *≤* *0.05 was considered significant. A multiple linear regression (multivariate analysis) was performed to identify factors associated with MOS. SPSS 19.0 (IBM, Chicago, IL) was used for the statistical analyses.

## Results

### Study selection and characteristics of eligible trials

**Figure 1:**
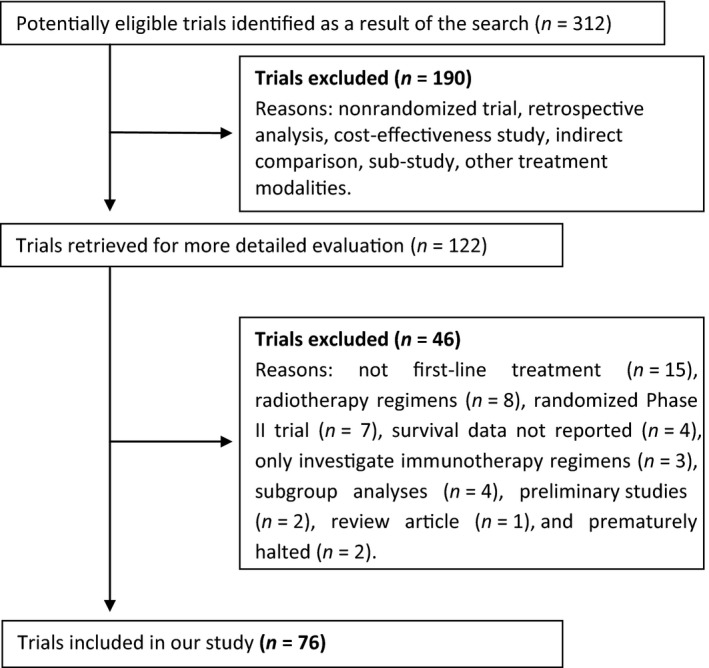
Flowchart depicting the process of study selection for the review.

As shown in the flowchart in Figure 1, out of 122 potentially eligible trials initially identified, 46 were excluded after application of the study eligibility criteria. The 76 trials finally selected in the review (a list of the 76 clinical trials is given as online supporting information) involved a total of 40,765 patients with advanced NSCLC randomly assigned to 152 arms, including six arms with best supportive care (BSC). The baseline characteristics of the selected trials are listed in Table 1.

**Table 1:** Characteristics of the 76 eligible trials (per trial)

Variable	*n* (%)
(No. of randomized patients in all trials 40,765)	
Year of trial publication	
2000	3 (3.9)
2001	5 (6.6)
2002	7 (9.2)
2003	7 (9.2)
2004	7 (9.2)
2005	7 (9.2)
2006	7 (9.2)
2007	6 (7.9)
2008	5 (6.6)
2009	4 (5.3)
2010	7 (9.2)
2011	5 (5.3)
2012	7 (9.2)
Sponsorship by industry	52 (68.4)
No. of randomized patients	
<500	46 (60.5)
501–1000	17 (22.4)
>1000	13 (17.1)
Median (range)	432 (126–1725)
Patients with a poor performance status^a^	44 (58.0)
Follow‐up, median (range),months Not recorded, n	17.6 (2.7–60)29
ITT analysis	51 (67.1)
Author′s study conclusion	
Experimental arm positive result	41 (53.9)
Experimental arm negative result	25 (32.9)
Experimental and control arm similar result	10 (13.2)

ITT, intention to treat.

a Defined as ECOG grade ≥2 or Karnofsky grade ≤60.

#### Sample size

The number of randomized patients per trial increased 14 patients per year of publication. Although this trend was not statistically significant (*P *=* *0.178), a significant increase in sample size was observed between 2008 and 2012 (*P *=* *0.032). The sample size was significantly higher in trials of molecular‐targeted versus nontargeted agents (740.8 vs. 460.1; *P *=* *0.009), in trials of more than one drug versus trials of a single drug (584.8 vs. 360.4; *P *=* *0.014), and in trials including patients with good performance status (ECOG<2 or KPS scale >60) versus those with poorer status (675.3 vs. 425.6; *P *=* *0.003). Conversely, the source of funding did not affect the sample size of the trial. From trials that involved targeted therapy (*n* = 19), only five were biomarker directed (population biomarker +) with a smaller sample size than trials that used a targeted agent indiscriminately in the entire population (biomarker + and biomarker −) (374.0 vs. 886.1; *P* = 0.034). The median IF of the journals in which the trials were published was 18.97 (range, 2.36–53.49) and increased 1.064 points per year of publication (*P *=* *0.005). The IF was significantly and positively correlated with the sample size (*r* = 0.377, *P *<* *0.001) and with the MOS (*r* = 0.302, *P *=* *0.009) and was negatively correlated with the median follow‐up time (*r* = −0.417, *P *=* *0.003), finding that studies with a shorter median follow‐up were published in journals with higher IF.

A total of 41 (53.9%) studies concluded that the result was positive, defined by the authors’ recommendation of adoption of the treatment or further studies. The mean (standard deviation) IF was higher for journals publishing trials that reported positive results versus journals publishing trials that did not (20.7 [15.4] vs. 14.0 [7.00], *P *=* *0.002).

#### QoL assessment

QoL was evaluated in 46 trials (60.5%). The specific Functional Assessment of Cancer Therapy Lung Cancer questionnaire (FACLT‐LCS5) was used in 23 of these trials (50%), the European Organization for Research and Treatment of Cancer Quality of Life questionnaire C30 (EORTC QLQ C‐30) in 20 trials (43.5%), and the specific EORTC lung cancer questionnaire (EORTC QLQ LC‐13) in 17 trials (37.0%).

### Trends in the types of chemotherapy arms

In total, 21 different agents were used across the 76 selected RCTs. Table 2 exhibits the 22 most frequently applied regimens, which represented 82% of the chemotherapy regimens recorded. Platinum‐based regimens were the most frequently investigated regimens throughout the study period and were used in at least one arm of the trial in 66 (86.8%) of the RCTs across 106 arms. Molecular‐targeted agents administered in combination with chemotherapy or alone were investigated in 19 trials (25.0%) across 19 arms.

**Table 2:** The 22 most frequently used regimens in the eligible trials

Regimen	No. of arms
Paclitaxel + Carboplatin	28
Gemcitabine + Cisplatin	20
Vinorelbine + Cisplatin	12
Docetaxel + Cisplatin	10
Gemcitabine + Carboplatin	9
Paclitaxel + Cisplatin	8
Gemcitabine	6
Gemcitabine + Paclitaxel	5
Gemcitabine + Vinorelbine	5
Vinorelbine	4
Erlotinib	4
Gefitinib	4
Docetaxel	4
Etoposide + Cisplatin	4
Docetaxel + Carboplatin	4
Gemcitabine + Docetaxel	4
Mitomycin C + Vindesine + Cisplatin	4
Mitomycin C + Ifosfamide + Cisplatin	3
Gemcitabine + Vinorelbine + Cisplatin	3
Irinotecan + Cisplatin	3
Paclitaxel + Gefitinib + Cisplatin	3
Gemcitabine + Gefitinib + Cisplatin	3

As expected, the percentage of trials that included molecular‐targeted agents increased with each year of publication, especially between 2008 and 2012 (8.2% vs. 53.6%. *P *<* *0.001).

### Trends in patient median overall survival and progression‐free survival

**Figure 2:**
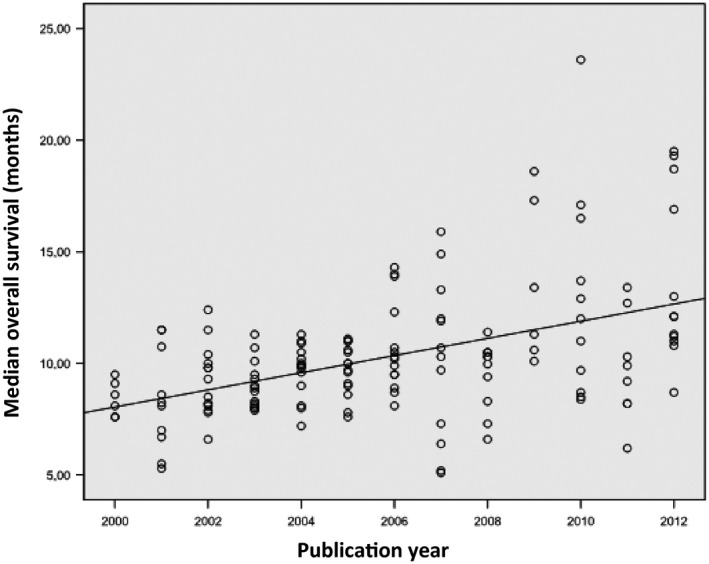
Relationship between year of trial publication and median survival time. Each trial is represented by a circle.

Table 3 displays the MOS reported for each type of chemotherapy regimen. In general, a survival benefit appeared to be obtained with newer agents, defined as third‐generation cytotoxic drugs (docetaxel, gemcitabine, irinotecan, paclitaxel, pemetrexed, and vinorelbine; 16 or with molecular‐targeted agents. OS data were available for 69 (90.8%) trials, and only 13 (18.8%) reported a statistically significant difference in OS between the arms, which was in favor of the experimental arm in 13 trials. The MOS for the 76 trials was 9.90 months (IQR = 3.5). Figure 2 depicts a scatter plot for the year of trial publication and MOS per arm, showing an increase in MOS of 0.384 months per year (*P *<* *0.001).

**Table 3:** Types of chemotherapy arms and treatment outcomes (per active treatment arm)

Chemotherapy arm	No. of arms (%)	MOS (IQR), months	MPFS (IQR), months
Total no. of arms	152	9.90 (3.5)	4.9 (1.9)
Platinum‐based regimen			
Combined with targeted agent^a^	11 (7.2)	11.3 (3.1)	5.5 (0.9)
Combined with newer agents^b^	78 (51.3)	10.0 (2.5)	4.9 (1.5)
Other combinations	17 (11.2)	8.6 (2.3)	3.7 (2.5)
Non‐platinum‐based regimens			
Targeted agent	8 (5.3)	18.7 (10.2)	7.8 (5.4)
Newer agent	29 (19.1)	9.4 (2.9)	3.8 (1.8)
Other combinations	3 (2.0)	11.3 (1.8)	7.5 (3.1)
BSC	6 (3.9)	10.5 (5.1)	2.5 (0.6)

MOS, median overall survival (months); MPFS, median progression‐free survival (months); IQR, interquartile range; BSC, best supportive care.

a Defined as agents acting on known specific molecular targets, such as epidermal growth factor receptor and angiogenesis pathway: gefitinib, erlotinib, cetuximab, bevacizumab, vadimezan (ASA404), and motesanib (AMG 706) (see Domvri et al., 2013).

b Defined as third‐generation cytotoxic drugs: docetaxel, gemcitabine, irinotecan, paclitaxel, pemetrexed, and vinorelbine (see Azzoli et al., 2009).

**Figure 3:**
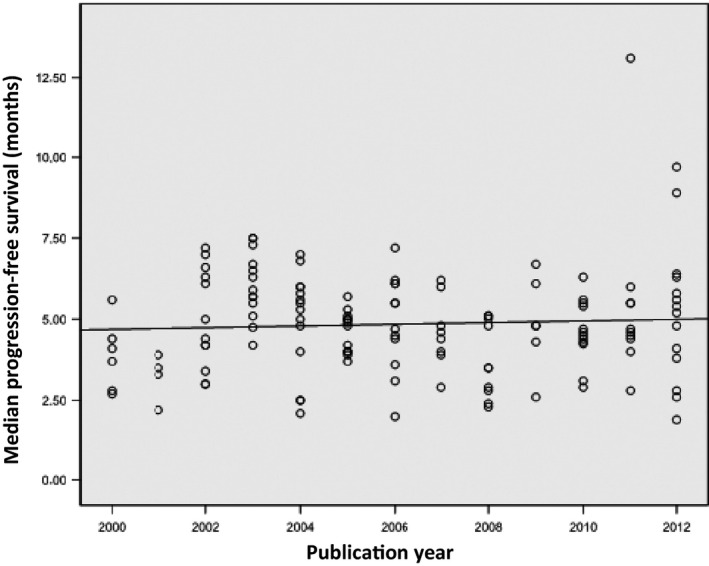
Relationship between year of trial publication and median progression‐free survival. Each trial is represented by a circle.

Progression‐free survival (PFS) data were available for 67 (88.2%) trials. A significant difference between arms was reported in 26 (38.8%) trials, in favor of the experimental arm in 24, and in favor of the control arm in the remaining 2. The MPFS was 4.90 months (IQR = 1.9). Figure 3 depicts a scatter plot for the year of trial publication and MPFS per arm, showing an increase in MPFS of 0.026 month per year (*P*=>0.05). A statistically significant correlation was found between the MPFS and MOS (r = 0.268; *P *=* *0.032).

#### Multivariate analysis (multiple linear regression)

Data are presented in Table 4. Among all variables studied, molecular‐targeted agents regimens (*P* = 0.018) and year of publication after 2008 (*P* = 0.047) were independent positive predictors for a longer MOS, whereas more than two drug‐based regimen (*P* = 0.002) was an independent negative predictor for longer MOS.

**Table 4:** Multivariate analyses of variables related with MOS (multiple linear regression)

Predictor	MOS		
	*β*	SE	*P*
Constant	4.685	4.372	0
Molecular‐targeted agents‐based regimens	2.355	0.964	0.018
Number of agents combined	−3.412	1.077	0.002
Significant increase in PFS	1.595	0.845	0.064
Platinum‐based regimen	3.57	1.323	0.009
Publication year	1.718	0.844	0.047

MOS, median overall survival; PFS, progression free survival.

QoL data were available for 46 trials (60.5%), and a statistically significant improvement was reported in 10 (21.7%) of these (not shown in tables). The scarcity of data on QoL outcomes prevented analysis of changes over the time period studied.

The percentage of trials reporting positive results has increased from 44.9% before 2008 to 67.9% after 2008, but this change is not statistically significant (*P* = 0.077).

## Discussion

This study of phase III RCTs on advanced NSCLC published between 2000 and 2012 revealed an increase in the size of these trials over time, especially after 2008, and a perceptible but modest improvement in patient outcomes.

The increase in MOS over this 12‐year period was 0.384 months per year *(*1.670 weeks/year, assuming 1 month = 4.348 weeks), which is higher than the increase reported in the review of phase III trials on NSCLC by Breathnach and Freidlin 17 between 1973 and 1994 (≈0.1 months per year) and in the review by Hotta et al. 18 between 1982 and 2002 (0.12 months per year). The greater increase in MOS observed in our more recent review may be attributable to the introduction of novel approaches to the treatment of this entity as indicated by the results from the multivariate analyses performed here, as well as the introduction of platinum‐based regimens. The discovery of different cancer‐related pathways has led to the development of targeted therapies (e.g., erlotinib, gefitinib, cetuximab) that have improved the prognosis and the MOS rates 8, 19. Further factors influencing this improvement may include the use of more selective inclusion criteria which leads to select patients with better prognosis, a more effective use of radiation therapy, better supportive care 18, inclusion of only subgroups of patients selected according to predictive biomarker for whom treatment is more effective or the earlier treatment of patients 20. Although the most frequently investigated treatments continued to be platinum‐based regimens, there was a significant increase in the percentage of RCTs on molecular‐targeted therapies (*P *<* *0.001), with 80% of these being published after 2008. MOS and MPFS outcomes (months) were superior in arms with a targeted agent in monotherapy than in those in which a targeted agent was combined with a platinum regimen (MOS: 18.7 vs. 11.3, MPFS: 7.8 vs. 5.5) and, among the former, they were better in patients selected for molecular EGFR mutation than in those who were not (MOS: 26.9 vs. 14.5; *P* = 0.057 and MPFS: 10.3 vs. 4.8). According to our findings, the addition of an EGFR tyrosine kinase inhibitor (78.3% of molecular target agents in this study) to chemotherapy offers no benefit in survival or PFS, supporting the updated clinical practice guidelines of the American Society of Clinical Oncology on chemotherapy for Stage IV non‐small‐cell lung cancer 21. In contrast, the administration of bevacizumab or cetuximab is recommended in combination with cytotoxic drugs 21.

The median (IQR) PFS for patients with advanced NSCLC over the study period was 4.9 (1.9) months, similar to the median of 5 months observed by Hotta et al. between 1996 and 2002. The increase between 2000 and 2012 of 0.026 months/year was even smaller than that observed by *Hotta* et al. (0.062 months/year). This suggests that the modest improvement in OS observed over the past decade was not accompanied by a corresponding increase in PFS. However, study of the targeted therapies (non‐platinum‐based regimens) alone reveals a greater improvement in PFS (median of 7.8 months). Interestingly, the MPFS in these trials was 10.3 months in those selecting EGFR mutation‐positive (EGFRmut+) patients versus 4.8 months in those that did not, which supports EGFR mutation assessment before treatment, considering EGFR‐TKIs as first‐line treatment in positive patients 22.

It is disturbing that only 13 (18.8%) of the 76 trials reviewed showed a statistically significant increase in OS, similar to the proportion reported in previous decades 17, 23. Despite these results, a positive conclusion was reached by the authors in 41 (53.9%) of the trials, which cannot be justified by a true increase in the PFS, which was only significantly improved in 26 (38.8%) of them. In fact, the percentage of studies reporting positive in their findings has increased. This discrepancy has previously been attributed to a lack of statistical rigor or to pharmaceutical industry sponsorship and the consequent pressure for a positive result 10, 23. Further research is warranted to study the correlation between favorable conclusions and different variables. Thus, there is ongoing controversy on the use of PFS as a primary study endpoint 10, 24, 25, which appears to be increasingly popular in oncology, probably due to the greater likelihood of finding a significant improvement in PFS than in OS.

Deficiencies were observed in the quality of some of the reviewed trials, including the absence of ITT analysis and a short median follow‐up 14, 23, 26. In earlier reviews, data on median follow‐up were only reported in 57% of articles on different tumor types 27 and in 48% of those on advanced breast cancer 28.

The QoL of patients has been found to be a strong predictor of survival and toxicity outcomes 29, 30, but this outcome was studied in only 60.5% of the present trials, similar to the proportion reported in a previous review of RCTs 31. Moreover, only 21.7% of these trials reported a significant improvement in QoL, although this is better than the finding by Tanvetyanon et al. in their 2007 review of chemotherapy in NSCLC, in which only 7.1% of trials showed a significant difference in QoL. Results have evidenced a modest improvement, but further research is needed on QoL in NSCLC patients, with greater uniformity in the methodologies employed 30.

Our review has some limitations. It was not always possible to obtain all of the data required to evaluate the methodology and outcomes of the studies. In addition, PFS and time to progression were considered as surrogate endpoints of survival and as interchangeable, in common with other authors 28. It has previously been reported that comparisons among clinical trials in oncology are hampered by a lack of consistency in the definition of efficacy outcomes 27, 28, 32. One major weakness of this study is the lack of information on side effects, due to the methodological difficulty of comparing among studies that use different scales and consider distinct toxicity outcomes. Finally, our aim was not to perform a meta‐analysis but rather to conduct a review of practical relevance for the clinician.

This critical analysis of the time course of clinical trials in NSCLC shows that a moderate but continuous improvement in the survival of patients with advanced NSCLC has been achieved over the past 12 years. Novel targets, specific strategic approaches, and improvements in the methodology and quality of trials will be essential in future research. Importantly, the discrepancy between the reported outcomes and the conclusions of some authors suggests the need for rigorous critical evaluation of the quality of the results of clinical trials before potentially costly changes are introduced into clinical practice. It is important to distinguish between a statistically significant improvement and a clinically meaningful benefit 33, and it is vital to elucidate the true cost‐effectiveness of newly adopted therapies. At present, research in this field is mostly focused on immunotherapy, and this study should be reproduced in a few years to compare benefits and quality of trials.

## Conflict of Interest

None declared.

## Supporting information


**Data S1.** A list of the 76 phase III clinical trials included in this review is available as online supplementary material.
