# Supplementary material for: Trends in phase III randomized controlled clinical trials on the treatment of advanced non‐small‐cell lung cancer
Source: Cancer Med. 2016 Jul 23;5(9):2190–7. doi: 10.1002/cam4.782 (PMC5055155; doi:10.1002/cam4.782)
Supplement: Supplementary file 1 — Data S1. A list of the 76 phase III clinical trials included in this review is available as online supplementary material. [file CAM4-5-2190-s001.pdf]

Trends in phase III randomized controlled clinical trials on the treatment of advanced non-small cell lung cancer

Journal of Supportive Care in Cancer

Author names and affiliations: C. Fernández-López[1], J. Expósito-Hernández[2], J.P. Arebola-Moreno[2], M.A. Calleja- Hernández[1], J. Cabeza-Barrera[1], M. Expósito-Ruíz[3], R. Guerrero- Tejada[2], I. Linares[2]

[1]Department of Pharmacy, Instituto de Investigación Biosanitaria ibs.GRANADA. Hospitales Universitarios de Granada/Universidad de Granada, Granada, Spain.

[2]Department of Oncology, Virgen de las Nieves University Hospital, Granada, Spain.

[3]University Hospital Research Unit; Instituto de Investigación Biosanitaria ibs,.GRANADA, Spain.

Corresponding author: Ms. Cristina Fernández-López, Department of Pharmacy, Instituto de Investigación Biosanitaria ibs.GRANADA. Hospitales Universitarios de Granada/Universidad de Granada, Granada, Spain. Calle Doctor Oloriz 16, Granada, 18012, Spain. Telephone: +34 619 756 566, E-mail: cristinafernandez85@gmail.com

Supplementary material

A list of the 76 phase III clinical trials included in this review is available as online supplementary material.

1. Gatzemeier U, von Pawel J, Gottfried M et al. Phase III comparative study of high-dose cisplatin versus a combination of paclitaxel and cisplatin in patients with advanced non-small-cell lung cancer. J. Clin. Oncol. 2000; 18(19):3390–9.

2. Sandler AB, Nemunaitis J, Denham C et al. Phase III trial of gemcitabine plus cisplatin versus cisplatin alone in patients with locally advanced or metastatic non-small-cell lung cancer. J. Clin. Oncol. 2000; 18(1):122–30.

3. Bonomi P, Kim K, Fairclough D et al. Comparison of survival and quality of life in advanced non-small-cell lung cancer patients treated with two dose levels of paclitaxel combined with cisplatin versus etoposide with cisplatin: results of an Eastern Cooperative Oncology Group trial. J. Clin. Oncol. 2000; 18(3):623–31.

4. Le Chevalier T, Brisgand D, Soria JC et al. Long term analysis of survival in the European randomized trial comparing vinorelbine/cisplatin to

vindesine/cisplatin and vinorelbine alone in advanced non-small cell lung cancer. Oncologist 2001; 6 Suppl 1:8–11.

5. Gridelli C. The ELVIS trial: a phase III study of single-agent vinorelbine as first-line treatment in elderly patients with advanced non-small cell lung cancer. Elderly Lung Cancer Vinorelbine Italian Study. Oncologist 2001; 6 Suppl 1:4–7.

6. Vansteenkiste JF, Vandebroek JE, Nackaerts KL et al. Clinical-benefit response in advanced non-small-cell lung cancer: A multicentre prospective randomised phase III study of single agent gemcitabine versus cisplatin-vindesine. Ann. Oncol. 2001; 12(9):1221–30.

7. Kelly K, Crowley J, Bunn PA et al. Randomized phase III trial of paclitaxel plus carboplatin versus vinorelbine plus cisplatin in the treatment of patients with advanced non--small-cell lung cancer: a Southwest Oncology Group trial. J. Clin. Oncol. 2001; 19(13):3210–8.

8. Fukuoka, M, Negoro, S, Masuda N et al. Irinotecan/Cisplatin versus Vindesine/Cisplatin versus Irinotecan Alone in Advanced Non–Small-Cell Lung Cancer. Clin. Lung Cancer 2001; 2(3):180–181.

9. Socinski MA, Schell MJ, Peterman A et al. Phase III trial comparing a defined duration of therapy versus continuous therapy followed by second-line therapy in advanced-stage IIIB/IV non-small-cell lung cancer. J. Clin. Oncol. 2002; 20(5):1335–43.

10. Kosmidis P, Mylonakis N, Nicolaides C et al. Paclitaxel plus carboplatin versus gemcitabine plus paclitaxel in advanced non-small-cell lung cancer: a phase III randomized trial. J. Clin. Oncol. 2002; 20(17):3578–85.

11. Souquet PJ, Tan EH, Rodrigues Pereira J et al. GLOB-1: a prospective randomised clinical phase III trial comparing vinorelbine-cisplatin with vinorelbine-ifosfamide-cisplatin in metastatic non-small-cell lung cancer patients. Ann. Oncol. 2002; 13(12):1853–61.

12. Rosell R, Gatzemeier U, Betticher DC et al. Phase III randomised trial comparing paclitaxel/carboplatin with paclitaxel/cisplatin in patients with advanced non-small-cell lung cancer: a cooperative multinational trial. Ann. Oncol. 2002; 13(10):1539–49.

13. Kodani T, Ueoka H, Kiura K et al. A phase III randomized trial comparing vindesine and cisplatin with or without ifosfamide in patients with advanced non-small-cell lung cancer: long-term follow-up results and analysis of prognostic factors. Lung Cancer 2002; 36(3):313–9.

14. Grigorescu AC, Draghici IN, Nitipir C et al. Gemcitabine (GEM) and carboplatin (CBDCA) versus cisplatin (CDDP) and vinblastine (VLB) in advanced non-small-

cell lung cancer (NSCLC) stages III and IV: a phase III randomised trial. Lung Cancer 2002; 37(1):9–14.

15. Schiller JH, Harrington D, Belani CP et al. Comparison of four chemotherapy regimens for advanced non-small-cell lung cancer. Nejm 2002; 346(2):92–8.

16. Wachters FM, Van Putten JWG, Kramer H et al. First-line gemcitabine with cisplatin or epirubicin in advanced non-small-cell lung cancer: a phase III trial. Br. J. Cancer 2003; 89(7):1192–9.

17. Zatloukal P, Petruzelka L, Zemanová M et al. Gemcitabine plus cisplatin vs. gemcitabine plus carboplatin in stage IIIb and IV non-small cell lung cancer: a phase III randomized trial. Lung Cancer 2003; 41(3):321–31.

18. Gridelli C, Gallo C, Shepherd FA et al. Gemcitabine plus vinorelbine compared with cisplatin plus vinorelbine or cisplatin plus gemcitabine for advanced non-small-cell lung cancer: a phase III trial of the Italian GEMVIN Investigators and the National Cancer Institute of Canada Clinical Trials . J. Clin. Oncol. 2003; 21(16):3025–34.

19. Alberola V, Camps C, Provencio M et al. Cisplatin plus gemcitabine versus a cisplatin-based triplet versus nonplatinum sequential doublets in advanced non-small-cell lung cancer: a Spanish Lung Cancer Group phase III randomized trial. J. Clin. Oncol. 2003; 21(17):3207–13.

20. Smit EF, van Meerbeeck JPAM, Lianes P et al. Three-arm randomized study of two cisplatin-based regimens and paclitaxel plus gemcitabine in advanced non-small-cell lung cancer: a phase III trial of the European Organization for Research and Treatment of Cancer Lung Cancer Group--EORTC 08975. J. Clin. Oncol. 2003; 21(21):3909–17.

21. Fossella F. Randomized, Multinational, Phase III Study of Docetaxel Plus Platinum Combinations Versus Vinorelbine Plus Cisplatin for Advanced Non-Small-Cell Lung Cancer: The TAX 326 Study Group. J. Clin. Oncol. 2003; 21(16):3016–3024.

22. Danson S, Middleton MR, O'Byrne KJ et al. Phase III trial of gemcitabine and carboplatin versus mitomycin, ifosfamide, and cisplatin or mitomycin, vinblastine, and cisplatin in patients with advanced nonsmall cell lung carcinoma. Cancer 2003; 98(3):542–53.

23. Stathopoulos GP, Veslemes M, Georgatou N et al. Front-line paclitaxel-vinorelbine versus paclitaxel-carboplatin in patients with advanced non-small-cell lung cancer: a randomized phase III trial. Ann. Oncol. 2004; 15(7):1048–55.

24. Paccagnella A, Favaretto A, Oniga F et al. Cisplatin versus carboplatin in combination with mitomycin and vinblastine in advanced non small cell lung

cancer. A multicenter, randomized phase III trial. Lung Cancer 2004; 43(1):83–91.

25. Laack E, Dickgreber N, Müller T et al. Randomized phase III study of gemcitabine and vinorelbine versus gemcitabine, vinorelbine, and cisplatin in the treatment of advanced non-small-cell lung cancer: from the German and Swiss Lung Cancer Study Group. J. Clin. Oncol. 2004; 22(12):2348–56.

26. Kubota K, Watanabe K, Kunitoh H et al. Phase III randomized trial of docetaxel plus cisplatin versus vindesine plus cisplatin in patients with stage IV non-small-cell lung cancer: the Japanese Taxotere Lung Cancer Study Group. J. Clin. Oncol. 2004; 22(2):254–61.

27. Herbst RS, Giaccone G, Schiller JH et al. Gefitinib in combination with paclitaxel and carboplatin in advanced non-small-cell lung cancer: a phase III trial--INTACT 2. J. Clin. Oncol. 2004; 22(5):785–94.

28. Georgoulias V, Ardavanis A, Agelidou A et al. Docetaxel versus docetaxel plus cisplatin as front-line treatment of patients with advanced non-small-cell lung cancer: a randomized, multicenter phase III trial. J. Clin. Oncol. 2004; 22(13):2602–9.

29. Giaccone G. Gefitinib in Combination With Gemcitabine and Cisplatin in Advanced Non-Small-Cell Lung Cancer: A Phase III Trial--INTACT 1. J. Clin. Oncol. 2004; 22(5):777–784.

30. Rudd RM, Gower NH, Spiro SG et al. Gemcitabine plus carboplatin versus mitomycin, ifosfamide, and cisplatin in patients with stage IIIB or IV non-small-cell lung cancer: a phase III randomized study of the London Lung Cancer Group. J. Clin. Oncol. 2005; 23(1):142–53.

31. Martoni A, Marino A, Sperandi F et al. Multicentre randomised phase III study comparing the same dose and schedule of cisplatin plus the same schedule of vinorelbine or gemcitabine in advanced non-small cell lung cancer. Eur. J. Cancer 2005; 41(1):81–92.

32. Herbst RS, Prager D, Hermann R et al. TRIBUTE: a phase III trial of erlotinib hydrochloride (OSI-774) combined with carboplatin and paclitaxel chemotherapy in advanced non-small-cell lung cancer. J. Clin. Oncol. 2005; 23(25):5892–9.

33. Georgoulias V, Ardavanis A, Tsiafaki X et al. Vinorelbine plus cisplatin versus docetaxel plus gemcitabine in advanced non-small-cell lung cancer: a phase III randomized trial. J. Clin. Oncol. 2005; 23(13):2937–45.

34. Sederholm C, Hillerdal G, Lamberg K et al. Phase III trial of gemcitabine plus carboplatin versus single-agent gemcitabine in the treatment of locally

advanced or metastatic non-small-cell lung cancer: the Swedish Lung Cancer Study Group. J. Clin. Oncol. 2005; 23(33):8380–8.

35. Pujol J-L, Breton J-L, Gervais R et al. Gemcitabine-docetaxel versus cisplatin-vinorelbine in advanced or metastatic non-small-cell lung cancer: a phase III study addressing the case for cisplatin. Ann. Oncol. 2005; 16(4):602–10.

36. Belani CP, Lee JS, Socinski MA et al. Randomized phase III trial comparing cisplatin-etoposide to carboplatin-paclitaxel in advanced or metastatic non-small cell lung cancer. Ann. Oncol. 2005; 16(7):1069–75.

37. Schuette W, Blankenburg T, Guschall W et al. Multicenter randomized trial for stage IIIB/IV non-small-cell lung cancer using every-3-week versus weekly paclitaxel/carboplatin. Clin. Lung Cancer 2006; 7(5):338–43.

38. Kudoh S, Takeda K, Nakagawa K et al. Phase III study of docetaxel compared with vinorelbine in elderly patients with advanced non-small-cell lung cancer: results of the West Japan Thoracic Oncology Group Trial (WJTOG 9904). J. Clin. Oncol. 2006; 24(22):3657–63.

39. Brodowicz T, Krzakowski M, Zwitter M et al. Cisplatin and gemcitabine first-line chemotherapy followed by maintenance gemcitabine or best supportive care in advanced non-small cell lung cancer: a phase III trial. Lung Cancer 2006; 52(2):155–63.

40. Booton R, Lorigan P, Anderson H et al. A phase III trial of docetaxel/carboplatin versus mitomycin C/ifosfamide/cisplatin (MIC) or mitomycin C/vinblastine/cisplatin (MVP) in patients with advanced non-small-cell lung cancer: a randomised multicentre trial of the British Thoracic Oncology Group. Ann. Oncol. 2006; 17(7):1111–9.

41. Sandler A, Gray R, Perry MC et al. Paclitaxel-carboplatin alone or with bevacizumab for non-small-cell lung cancer. N. Engl. J. Med. 2006; 355(24):2542–50.

42. Park JO, Kim S-W, Ahn JS et al. Phase III trial of two versus four additional cycles in patients who are nonprogressive after two cycles of platinum-based chemotherapy in non small-cell lung cancer. J. Clin. Oncol. 2007; 25(33):5233–9.

43. Novello S, Bruzzi P, Barone C et al. Phase III study in stage IV non-small-cell lung cancer patients treated with two courses of cisplatin/gemcitabine followed by a randomization to three additional courses of the same combination or gemcitabine alone. Ann. Oncol. 2007; 18(5):903–8.

44. Helbekkmo N, Sundstrøm SH, Aasebø U et al. Vinorelbine/carboplatin vs gemcitabine/carboplatin in advanced NSCLC shows similar efficacy, but different impact of toxicity. Br. J. Cancer 2007; 97(3):283–9.

45. Greco FA, Spigel DR, Kuzur ME et al. Paclitaxel/Carboplatin/gemcitabine versus gemcitabine/vinorelbine in advanced non-small-cell lung cancer: a phase II/III study of the Minnie Pearl Cancer Research Network. Clin. Lung Cancer 2007; 8(8):483–7.

46. Hainsworth JD, Spigel DR, Farley C et al. Weekly docetaxel versus docetaxel/gemcitabine in the treatment of elderly or poor performance status patients with advanced nonsmall cell lung cancer: a randomized phase 3 trial of the Minnie Pearl Cancer Research Network. Cancer 2007; 110(9):2027–34.

47. Sculier JP, Lafitte JJ, Lecomte J et al. A phase III randomised trial comparing sequential chemotherapy using cisplatin-based regimen and paclitaxel to cisplatin-based chemotherapy alone in advanced non-small-cell lung cancer. Ann. Oncol. 2007; 18(6):1037–42.

48. Ohe Y, Ohashi Y, Kubota K et al. Randomized phase III study of cisplatin plus irinotecan versus carboplatin plus paclitaxel, cisplatin plus gemcitabine, and cisplatin plus vinorelbine for advanced non-small-cell lung cancer: Four-Arm Cooperative Study in Japan. Ann. Oncol. 2007; 18(2):317–23.

49. Comella P, Filippelli G, De Cataldis G et al. Efficacy of the combination of cisplatin with either gemcitabine and vinorelbine or gemcitabine and paclitaxel in the treatment of locally advanced or metastatic non-small-cell lung cancer: a phase III randomised trial of the Southern Italy Cooperative On. Ann. Oncol. 2007; 18(2):324–30.

50. Johnson EA, Marks RS, Mandrekar SJ et al. Phase III randomized, double-blind study of maintenance CAI or placebo in patients with advanced non-small cell lung cancer (NSCLC) after completion of initial therapy (NCCTG 97-24-51). Lung Cancer 2008; 60(2):200–7.

51. Georgoulias V, Androulakis N, Kotsakis A et al. Docetaxel versus docetaxel plus gemcitabine as front-line treatment of patients with advanced non-small cell lung cancer: a randomized, multicenter phase III trial. Lung Cancer 2008; 59(1):57–63.

52. O'Brien MER, Socinski MA, Popovich AY et al. Randomized phase III trial comparing single-agent paclitaxel Poliglumex (CT-2103, PPX) with single-agent gemcitabine or vinorelbine for the treatment of PS 2 patients with chemotherapy-naïve advanced non-small cell lung cancer. J. Thorac. Oncol. 2008; 3(7):728–34.

53. Scagliotti GV, Parikh P, von Pawel J et al. Phase III study comparing cisplatin plus gemcitabine with cisplatin plus pemetrexed in chemotherapy-naive patients with advanced-stage non-small-cell lung cancer. J. Clin. Oncol. 2008; 26(21):3543–51.

54. Kosmidis P a., Kalofonos HP, Christodoulou C et al. Paclitaxel and gemcitabine versus carboplatin and gemcitabine in patients with advanced non-small-cell lung cancer. A phase III study of the Hellenic Cooperative Oncology Group. Ann. Oncol. 2008; 19(1):115–122.

55. Mok TS, Wu Y-L, Thongprasert S et al. Gefitinib or carboplatin-paclitaxel in pulmonary adenocarcinoma. N. Engl. J. Med. 2009; 361(10):947–57.

56. Takeda K, Hida T, Sato T et al. Randomized Phase III Trial of Platinum-Doublet Chemotherapy Followed by Gefitinib Compared With Continued Platinum-Doublet Chemotherapy in Japanese Patients With Advanced Non-Small-Cell Lung Cancer: Results of a West Japan Thoracic Oncology Group Trial (W. J. Clin. Oncol. 2009; 28(5):753–760.

57. Reck M, von Pawel J, Zatloukal P et al. Phase III trial of cisplatin plus gemcitabine with either placebo or bevacizumab as first-line therapy for nonsquamous non-small-cell lung cancer: AVAil. J. Clin. Oncol. 2009; 27(8):1227–34.

58. Pirker R, Pereira JR, Szczesna A et al. Cetuximab plus chemotherapy in patients with advanced non-small-cell lung cancer (FLEX): an open-label randomised phase III trial. Lancet 2009; 373(9674):1525–31.

59. Ciuleanu T, Brodowicz T, Zielinski C et al. Maintenance pemetrexed plus best supportive care versus placebo plus best supportive care for non-small-cell lung cancer: a randomised, double-blind, phase 3 study. Lancet 2009; 374(9699):1432–40.

60. Yang C-H, Simms L, Park K et al. Efficacy and Safety of Cisplatin/Pemetrexed Versus Cisplatin/Gemcitabine as First-Line Treatment in East Asian Patients with Advanced Non-small Cell Lung Cancer. J. Thorac. Oncol. 2010; 5(5):1.

61. Mitsudomi T, Morita S, Yatabe Y et al. Gefitinib versus cisplatin plus docetaxel in patients with non-small-cell lung cancer harbouring mutations of the epidermal growth factor receptor (WJTOG3405): an open label, randomised phase 3 trial. Lancet. Oncol. 2010; 11(2):121–8.

62. Maemondo M, Inoue A, Kobayashi K et al. Gefitinib or chemotherapy for non-small-cell lung cancer with mutated EGFR. N. Engl. J. Med. 2010; 362(25):2380–8.

63. Lynch TJ, Patel T, Dreisbach L et al. Cetuximab and first-line taxane/carboplatin chemotherapy in advanced non-small-cell lung cancer: results of the randomized multicenter phase III trial BMS099. J. Clin. Oncol. 2010; 28(6):911–7.

64. Cappuzzo F, Ciuleanu T, Stelmakh L et al. Erlotinib as maintenance treatment in advanced non-small-cell lung cancer: a multicentre, randomised, placebo-controlled phase 3 study. Lancet. Oncol. 2010; 11(6):521–9.

65. Treat JA, Gonin R, Socinski MA et al. A randomized, phase III multicenter trial of gemcitabine in combination with carboplatin or paclitaxel versus paclitaxel plus carboplatin in patients with advanced or metastatic non-small-cell lung cancer. Ann. Oncol. 2010; 21(3):540–7.

66. Quoix E, Zalcman G, Oster J-P et al. Carboplatin and weekly paclitaxel doublet chemotherapy compared with monotherapy in elderly patients with advanced non-small-cell lung cancer: IFCT-0501 randomised, phase 3 trial. Lancet 2011; 378(9796):1079–88.

67. Zhou C, Wu Y-L, Chen G et al. Erlotinib versus chemotherapy as first-line treatment for patients with advanced EGFR mutation-positive non-small-cell lung cancer (OPTIMAL, CTONG-0802): a multicentre, open-label, randomised, phase 3 study. Lancet. Oncol. 2011; 12(8):735–42.

68. Lara PN, Douillard J-Y, Nakagawa K et al. Randomized phase III placebo-controlled trial of carboplatin and paclitaxel with or without the vascular disrupting agent vadimezan (ASA404) in advanced non-small-cell lung cancer. J. Clin. Oncol. 2011; 29(22):2965–71.

69. Weissman CH, Reynolds CH, Neubauer MA et al. A Phase III Randomized Trial of Gemcitabine–Oxaliplatin versus Carboplatin–Paclitaxel as First-Line Therapy in Patients with Advanced Non-small Cell Lung Cancer. J. Thorac. Oncol. 2011; 6(2):1.

70. Pérol M, Chouaid C, Pérol D et al. Randomized, phase III study of gemcitabine or erlotinib maintenance therapy versus observation, with predefined second-line treatment, after cisplatin-gemcitabine induction chemotherapy in advanced non-small-cell lung cancer. J. Clin. Oncol. 2012; 30(28):3516–24.

71. Scagliotti G V, Vynnychenko I, Park K et al. International, randomized, placebo-controlled, double-blind phase III study of motesanib plus carboplatin/paclitaxel in patients with advanced nonsquamous non-small-cell lung cancer: MONET1. J. Clin. Oncol. 2012; 30(23):2829–36.

72. Gridelli C, Ciardiello F, Gallo C et al. First-line erlotinib followed by second-line cisplatin-gemcitabine chemotherapy in advanced non-small-cell lung cancer: the TORCH randomized trial. J. Clin. Oncol. 2012; 30(24):3002–11.

73. Socinski MA, Bondarenko I, Karaseva NA et al. Weekly nab-paclitaxel in combination with carboplatin versus solvent-based paclitaxel plus carboplatin as first-line therapy in patients with advanced non-small-cell lung cancer: final results of a phase III trial. J. Clin. Oncol. 2012; 30(17):2055–62.

74. Zhang L, Ma S, Song X et al. Gefitinib versus placebo as maintenance therapy in patients with locally advanced or metastatic non-small-cell lung cancer (INFORM; C-TONG 0804): a multicentre, double-blind randomised phase 3 trial. Lancet. Oncol. 2012; 13(5):466–75.

75. Rosell R, Carcereny E, Gervais R et al. Erlotinib versus standard chemotherapy as first-line treatment for European patients with advanced EGFR mutation-positive non-small-cell lung cancer (EURTAC): a multicentre, open-label, randomised phase 3 trial. Lancet Oncol. 2012; 13(3):239–46.

76. Paz-Ares L, de Marinis F, Dediu M et al. Maintenance therapy with pemetrexed plus best supportive care versus placebo plus best supportive care after induction therapy with pemetrexed plus cisplatin for advanced non-squamous non-small-cell lung cancer (PARAMOUNT): a double-blind, phase 3, random. Lancet. Oncol. 2012; 13(3):247–55.
